# INNOVATIONS IN HOME MODIFICATION RESEARCH: THE STATE OF THE ART

**DOI:** 10.1093/geroni/igz038.2365

**Published:** 2019-11-08

**Authors:** Emily Nabors, Mindy Renfro, Jon Pynoos, Sarah L Szanton, Jon Sanford, Susan Stark

**Affiliations:** 1 University of Southern California, Los Angeles, California, United States; 2 Touro University Nevada, Henderson, Nevada, United States; 3 Johns Hopkins School of Nursing, Baltimore, Maryland, United States; 4 Georgia Tech, Atlanta, Georgia, United States; 5 Washington University School of Medicine in St. Louis, St. Louis, Missouri, United States

## Abstract

The overwhelming preference of older adults is to stay in their homes for as long as possible (AARP). However, most housing lacks supportive features and presents barriers that jeopardize residents’ ability to successfully age in place. Only 1% of houses have five key features to ensure accessibility: no-step entry, single-floor living, lever door handles, accessible electrical controls, and extra-wide doors and hallways (Harvard Joint Center for Housing Studies), making the vast majority unsuitable for persons who use wheelchairs and problematic for the growing number of people with activity limitations. Persons least likely to have such features in their homes need them the most: old-old, low income, frail, and residents in older housing stock. Although home modification can support people as their needs change and preclude the need to move, often to institutional settings, the majority of older adults lack these supports. Recent studies have demonstrated the role of home modification in health, safety, and cost effectiveness. This symposium will convene a panel of researchers to share evidence-base in home modification, recent cost-saving innovations including the CAPABLE Program, and policy change to improve service delivery.

